# First molecular detection of the genus Almendravirus in *Johnbelkinia ulopus* and *Anopheles apicimacula* mosquitoes from the Colombian Caribbean

**DOI:** 10.1186/s13071-026-07432-y

**Published:** 2026-07-19

**Authors:** Héctor Contreras-Martinez, Daniel Echeverri-De la Hoz, Yesica López, Yeimi López, Richard Hoyos, Luis Romero, Maira Alemán, Caty Martínez, Bertha Gastelbondo, Keren Álvarez, Gabriela Borja, Ketty Galeano, Alejandra García, Pedro Fragoso, German Arrieta, Salim Mattar

**Affiliations:** 1 Universidad de Córdoba, Montería, Córdoba Colombia; 2 Corporación Universitaria del Caribe (CECAR), Sincelejo, Sucre Colombia; 3 Universidad de Santander, Valledupar, Colombia; 4 Universidad Popular del Cesar, Valledupar, Cesar Colombia; 5 Universidad de Sucre, Sincelejo, Sucre Colombia

**Keywords:** Arbovirus, Metagenomic, Vector borne diseases, Emerging viruses, Insect-specific viruses, *Rhabdoviridae*

## Abstract

**Background:**

*Rhabdoviridae* includes many viruses, among which rabies virus is notable. Other genera in this family can infect mammals, birds, reptiles, fish, and plants.

**Methods:**

Between October 2022 and July 2023, mosquitoes were collected from some municipalities in the Córdoba and Cesar departments, Colombian Caribbean. Pools were formed according to taxonomic identification and geographic area. RNA was extracted, and sequencing was performed using MGI-G50 platform. Bioinformatics analyses were performed using the Galaxy platform and the Diamond-MEGAN program. The MAFFT program was used for sequence alignment. The Prokka program was used for genome annotation, IQ-TREE was used for phylogenetic reconstruction, and iTOL was used to visualize and edit the tree. The Clustal Omega program of the European Molecular Biology Laboratory (EMBL-EBI) was used to construct a percent similarity matrix, and Unipro UGENE was used to align the amino acids of the L protein with the conserved consensus sequence (GDNQ).

**Results:**

Two new genomes showing high similarity to *Almendravirus arboretum* (ABTV), and *Almendravirus chico* (RCHV) were identified in a single pool of *Johnbelkinia ulopus* mosquitoes collected in Córdoba. Additionally, a third genome with a low similarity percentage to the L segment of the *Almendravirus menghai* (MRV) from China was detected in *Anopheles apicimacula* from Cesar.

**Conclusions:**

This is the first study in Colombia that reports the ABTV and RCHV in *Jb. ulopus* mosquitoes and the first report of a phylogenetically similar sequence to the MRV, which could be a new virus of the *Rhabdoviridae* family.

**Graphical Abstract:**

**Figure:**
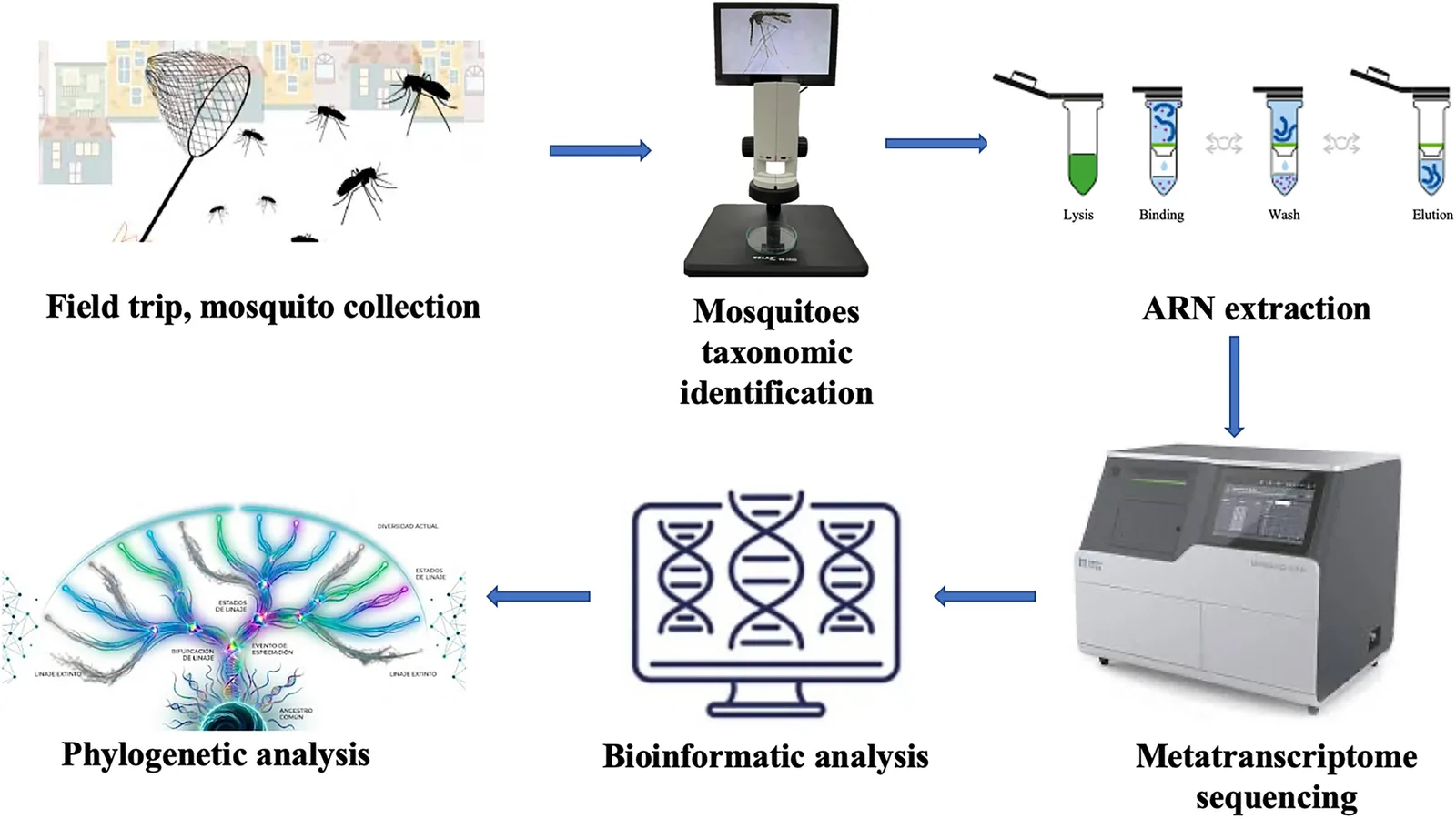

## Background

Mosquitoes are important for public health because they can transmit several viruses that infect humans and animals [1]. However, the natural viral community in mosquitoes also comprises other viruses, such as insect-specific viruses (ISVs) and those that infect symbiotic microorganisms [2, 3]. There has been an increase in studies on the mosquito virome, most of which have focused on urban mosquito species [4, 5]. However, little is known about the virome of wild mosquitoes, which contains more information on viral diversity and potentially emerging viruses.

Insect-specific viruses are essential because they can modify the susceptibility of mosquito vectors to pathogenic viruses [6]. However, some ISVs have been observed to favor pathogenic viruses; therefore, they require special attention because of their ability to alter the evolutionary potential of probable viral agents that can cause infections in humans [7]. Studies on new genomic sequences of disease-causing viruses transmitted by vectors, eco-epidemiology, and the interaction of ISVs are necessary, considering the modulation of pathogenicity and transmissibility in these interactions. It is also necessary to understand and study the implications of new viruses at an evolutionary level and their potential transmission to humans [3].

Members of the *Rhabdoviridae* family belong to the order Mononegavirales. In 2009, the International Committee on Taxonomy of Viruses (ICTV) recognized 6 genera and 51 species in this family. By 2024, the number of genera and species had increased to 56 and 434, respectively. They possess a negative-sense single-stranded RNA genome, and their genomes contain at least five genes that encode nucleoprotein (N), phosphoprotein (P), matrix protein (M), glycoprotein (G), and dependent RNA polymerase (L) in the ‘‘5’ direction [8]. Viruses that infect invertebrates, vertebrates, plants, and protozoa have been described within the Rhabdoviridae family [9, 10]. *Rhabdoviridae* comprises three subfamilies: *Alpharhabdovirinae*, *Betarhabdovirinae,* and *Gammarhabdovirinae.* Several divergent viral genera have not yet been assigned to subfamilies. *Alpharhabdovirinae* infect vertebrates and invertebrates, *Betarhabdovirinae* infect plants and are transmitted by arthropod vectors, and *Gammarhabdovirinae* infect fish [10]. Replicates in arthropods, such as mosquitoes, sandflies, and ticks, and can serve as vectors for human and animal infections [11, 12]. Within the family *Rhabdoviridae*, the genus *Almendravirus* was first proposed in 2017 as a new genus of *Rhabdovirus* isolated from mosquitoes. *Rhabdoviridae* has a genome size of 10.8–11.9 kb [13, 14], and in addition to the five proteins it encodes (N, P, M, G, and L), it also has a small hydrophobin (U1) between the G and L genes. The U1 protein in the viral genome has unique characteristics that have been used to identify almendraviruses [13] isolated in North America, South America, and Asia [13, 15].

From a One Health perspective, ISVs are essential because of the genetic variations and eco-epidemiological characteristics associated with climate change [16]. These factors may play a crucial role in the increased frequency of arbovirus emergence and their impact on host–vector–pathogen dynamics [17].

*An. apicimacula* are widely distributed and are considered secondary vectors of local importance in some regions. However, this species is not of epidemiological relevance in Colombia [18], whereas *Jb. ulopus* has been mentioned as a potential vector of arbovirus [19, 20]. Investigating viral diversity in these species is essential for understanding the epidemiological landscape of vector-borne diseases in the Caribbean and for the early identification of emerging viruses with zoonotic potential.

This study aimed to describe novel genomic sequences of the genus *Almendravirus* (*Rhabdoviridae*) detected through metagenomic and phylogenetic analyses in *Jb. ulopus* and *An. apicimacula* from Caribbean Colombia.

## Methods

### Type and area of study

**Figure 1:**
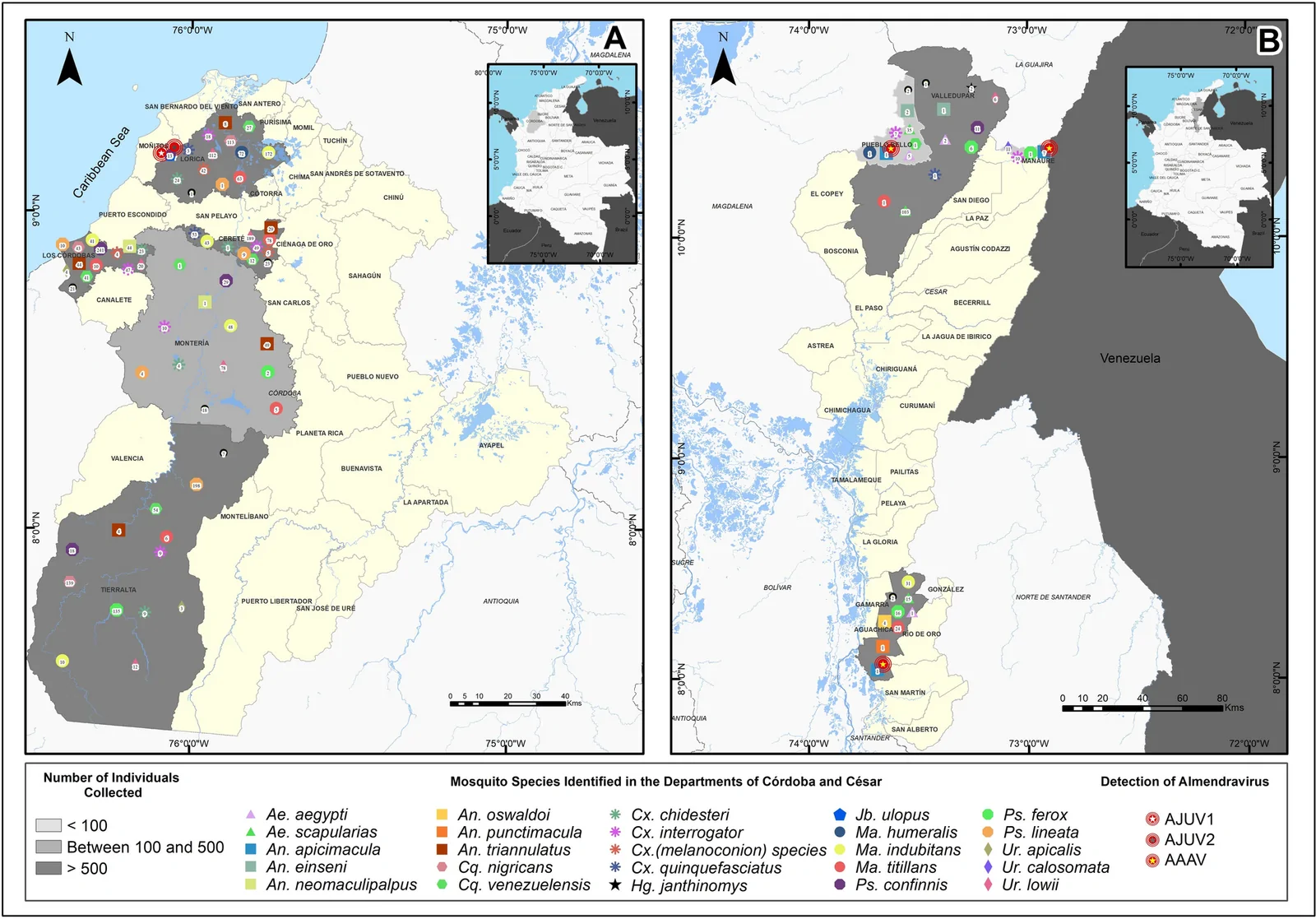
Mosquito species collected and viruses found in the departments of Córdoba and Cesar. **A** Map of the departments of Córdoba showing the samplings location and individuals number catch for location. **B** Map of the departments of Cesar showing the samplings location and individuals number catch for location

A prospective descriptive study was conducted as part of entomological surveillance of emerging vector-borne pathogens between October 2022 and July 2023. This study was conducted mainly in tropical dry forest areas of the departments of Córdoba and Cesar, located in the Colombian Caribbean (Fig. 1). In the department of Córdoba, captures were made in rural areas of the municipalities of Cerete, Lorica, Los Cordobas, Tierralta, Monteria, and Moñitos. The department of Córdoba has an average annual temperature of 28 °C and average annual rainfall of 2750 mm. In the department of Cesar, captures were made in rural areas of Valledupar, Pueblo Bello, Manaure, and Aguachica. The department of Cesar has an average temperature of 27.3 °C and average annual rainfall of 1500 mm. Three sampling events were conducted in both departments during the rainy and dry seasons. The municipalities were chosen on the basis of their size and agricultural activities. Ecological characteristics were considered, such as vegetation areas with little human intervention, crops, pastures, corrals with domestic animals, and water bodies. The sites chosen for entomological collection were georeferenced, and the perimeter and radius indices were estimated and used to construct the perimeter/area indices. Sampling was carried out under the permits of the National Authority of Environmental Licenses (ANLA), resolution no. 00914.

### Capturing, transporting, preserving, identifying, and forming mosquito pools

Adult female and male mosquitoes of different genera were collected from rural areas distant from the participants’ homes. Centers for Disease Control and Prevention (CDC) traps were used between 18:00 and 6:00 h, and manual captures were made with entomological nets and manual suction pumps between 7:00 and 18:30 h [21]. The captured specimens were separated from non-Culicid insects and placed in 1.5–2.0 ml tubes, then stored in tanks with liquid N2 and transported to the Institute of Biological Research in the Tropics (IIBT) of the University of Córdoba. Once in the laboratory, they were stored at −80 °C until identification [22–24]. The identified females were separated into 1.5 mL tubes per municipality to form pools of 50 individuals, which were stored at −80 °C until nucleic acid extraction.

### RNA extraction, library preparation, and sequencing

Female mosquito pools were macerated in 200 μl of Phosphate Buffer Saline 1X [PBS], then 400 μl of 1X PBS was added to make 600 μl, homogenized in a vortex, and centrifuged at 3000 g for 1 min. Subsequently, 200 μL was filtered through a 22-μm membrane, and nucleic acids were extracted from the filtrate using a GeneJET RNA purification kit (Thermo Fisher Scientific, Vilna, Lithuania). The extracted RNA was resuspended in RQ1 RNase-free Ô DNase (Promega). For sequencing, RNA pools were prepared according to the mosquito species and geographic areas. RNA concentration and integrity number (RIN) were measured by fluorometry using a QubitTM Broad Range (BR) RNA Quantification Kit and an RNA IQTM Assay Kit (Thermo Fisher Scientific, Eugene, Oregon, USA), respectively. A total of 250–500 ng of total RNA was used for library preparation. Samples were processed using the DNBSEQ-G50RS high-throughput sequencing kit (model: FCL PE150; MGI Technology, Wuhan, China), and the first and second DNA strands were synthesized using random hexamer primers. The fragments were subjected to end-catalysis repair (ERAT), molecular barcode ligation, and product amplification using a polymerase chain reaction (PCR). The library concentration was determined using the QubitTM dsDNA Quantification Assay Kit, and the fragment size was determined using a Fragment Analyzer^™^ (Agilent Technologies). Pools were obtained for DNA circularization and  DNA Nanoball (DNB) synthesis (> 11 ng/μl) on the basis of the fragment concentration and size. Next-generation sequencing was performed using DNB on MGI-G50TM equipment (Shenzhen, China).

### Bioinformatics analysis

Low-quality sequences (Phred score < Q20), short reads (less than 15 bp), and adapter sequences were removed using the Galaxy platform [25], using fastp v.0.23.2 [26], and host genome sequences for sequences from *Anopheles apicimacula* and *Johnbelkinia ulopus* were discarded by reference mapping using *Anopheles atroparvus* (GCA_000473505.1_Anop_atro_EBRO_V1) and *Sabethes cyaneus* (GCF_943734655.1_idSabCyanKW18_F2) reference sequences, respectively, in Bowtie2 v.2.5.0 [27], and de novo assembly was performed to obtain viral contigs or sequences using MEGAHIT v.1.2.9 [28]. Finally, taxonomic assignment was performed with DIAMOND v.2.1.9 [29] using the GenBank amino acid database to optimize the search for cds regions encoding viral proteins [30]. A pairing with a *P*-value of < 0.001 was optimized. The results were visualized and analyzed using MEGAN6 [30]. Genome annotation was performed using Prokka v.1.14.6 [31], and the predicted open reading frames (ORFs) were manually inspected and edited using Unipro UGENE v.50.0 [32]. This integrated approach ensured that highly divergent regions were correctly identified without the removal of ambiguous sequences.

### Genomic analysis

The obtained contigs were compared and confirmed using the NCBI non-redundant (nr) database with BLASTp and BLASTx [30]. Reference genome mapping was performed using Bowtie2 to obtain the average depth and coverage. Genomic comparisons and percentage similarity analyses were performed using SimPlot +  + v.3.5.1 [33]. Sequence alignment was performed using MAFFT [34] with sequences obtained from the GenBank database [35]. Phylogenetic reconstruction was performed using IQ-TREE v2.2.2.6 [36] and the maximum likelihood method [37]. Trees were reconstructed using 1000 replicates and visualized and edited using iTOL v5 [38]. In addition, a percent similarity matrix was created using reference amino acid sequences of the *Almendravirus* genus related to those obtained in this study, using Clustal Omega of the European Bioinformatics Institute (EMBL-EBI) [39] and edited in Excel [40]. The alignment of amino acids of the L protein (RdRp of negative ssRNA viruses with nonsegmented genomes catalytic domain profile) with the conserved consensus sequence (GDNQ) was carried out using Unipro UGENE v.50.0 [32].

## Results

To provide an overview of the entomological sampling results, 3026 mosquitoes were collected in Córdoba, of which 2757 were included in the sequencing pools, representing 7 genera and 18 species. The most frequent species were *Uranotaenia lowii* (14.6%, 411/2807) and *Mansonia indubitans* (10.9%, 305/2807). In Cesar, 364 mosquitoes were captured, of which 312 were selected for sequencing and grouped into pools covering 8 genera and 18 species. The most abundant species were *Aedes scapularis* (49%, 153/312) and *Mansonia indubitans* (9.9%, 40/312), followed by *Culex* (Table 1).

**Table 1:** Taxonomic composition and sampling characteristics of mosquito pools included in the analysis

Species	No. of ind	N. Ps	Municipality	Collection method	Capture season
*Ae. aegypti*	8	4	Aguachica, Pueblo Bello, Valledupar	AS/LT	RS,DS,TS
*Ae. scapularias*	153	8	Aguachica, Pueblo Bello, Valledupar	AS/LT	RS,DS,TS
*An. apicimacula*	9	4	Aguachica, Manaure, Pueblo Bello	AS/LT	RS,DS,TS
*An. eiseni*	3	3	Pueblo Bello, Valledupar	AS/LT	DS,TS
*An. neomaculipalpus*	45	3	Los Cordobas, Monteria	AS/LT	RS,DS
*An. oswaldoi*	4	1	Aguachica	AS/LT	DS
*An. punctimacula*	1	1	Aguachica	AS/LT	DS
*An. triannulatus*	134	10	Cerete, Lorica, Los Cordobas, Monteria, Tierralta	AS/LT	RS,DS,TS
*Cq. nigricans*	295	11	Lorica, Los Cordobas, Tierralta	AS/LT	RS,DS,TS
*Cq. venezuelensis*	136	9	Cerete, Lorica, Los Cordobas, Monteria, Pueblo Bello, Tierralta	AS/LT	RS,DS,TS
*Cx. chidesteri*	61	9	Cerete, Lorica, Los Cordobas, Monteria, Tierralta	AS/LT	RS,DS,TS
*Cx. interrogator*	150	16	Cerete, Lorica, Los Córdobas, Manaure, Monteria, Pueblo Bello, Tierralta	AS/LT	RS,DS,TS
*Cx. (melanoconion) species*	51	5	Cerete, Lorica, Los Córdobas	AS/LT	RS,DS,TS
*Cx. quinquefasciatus*	57	5	Cerete, Lorica, Valledupar	AS/LT	DS,TS,
*Hg. janthinomys*	2	2	Valledupar	AS/LT	DS,TS,
*Ma. humeralis*	73	3	Lorica, Pueblo Bello	AS/LT	DS,TS
*Ma. indubitans*	345	13	Aguachica, Cerete, Lorica, Los Cordobas, Monteria, Tierralta	AS/LT	RS,DS,TS
*Ma. titillans*	187	15	Aguachica, Cerete, Lorica, Los Cordobas, Monteria, Tierralta, Valledupar	AS/LT	RS,DS,TS
*Ps. confinnis*	299	11	Los Cordobas, Monteria, Tierralta, Valledupar	AS/LT	RS,DS,TS
*Ps. ferox*	160	11	Aguachica, Manaure, Monteria, Tierralta, Valledupar	AS/LT	RS,DS,TS
*Ps. lineata*	222	10	Cerete, Lorica, Los Cordobas, Monteria, Tierralta	AS/LT	RS,DS,TS
*Ur. apicalis*	241	8	Lorica, Los Cordobas, Tierralta	AS/LT	RS,DS,TS
*Ur. calosomata*	11	1	Manaure,	AS/LT	DS
*Ur. lowii*	411	17	Cerete, Lorica, Los Cordobas, Monteria, Tierralta,Valledupar	AS/LT	RS,DS,TS
*Jb. ulopus*	11	1	Moñitos	AS	TS

Capture methods: active search (AS), light trap (LT); capture season: rainy season (RS) October and November, dry season (DS) January–March, transition season (TS) April and May

Bioinformatics analyses revealed two distinct genomes of the *Almendravirus* genus co-occurring within the same pool of *Jb. ulopus* (11 individuals) from Córdoba, Colombia. This natural co-infection involved *Almendravirus Jb. ulopus* 1 (AJUV 1) and *Almendravirus Jb. ulopus* 2 (AJUV 2), which are closely related to ABTV and RCHV, respectively. In the department of Cesar, a sequence with low similarity to the gene encoding the structural L protein of the *Rhabdoviridae* family was found in *An. apicimacula*. The *An*. *apicimacula* species consisted of nine specimens collected from the municipalities of Aguachica 1/9 (11.1%), Manaure 7/9 (77.78%), and Pueblo Bello 1/9 (11.1%) (Fig. 1).

Bioinformatics analyses revealed two genomes of the *Almendravirus* genus in *Jb. ulopus* from Córdoba, Colombia, namely *Almendravirus Jb. ulopus* 1 (AJUV), which is 11,631 bp long and corresponds to 34,974 reads from 32.196.528 (0.11%), with an average read depth of 448X, and *Almendravirus Jb. ulopus* 2 (AJUV2), which is 11,128 bp long and corresponds to 9928 reads from 32.196.528 (0.031%), with an average read depth of 133X. The two new genomes were related to ABTV Peru (GenBank: KC994644.1) and RCHV Panamá (GenBank: NC_031958.1), respectively.

**Figure 2:**
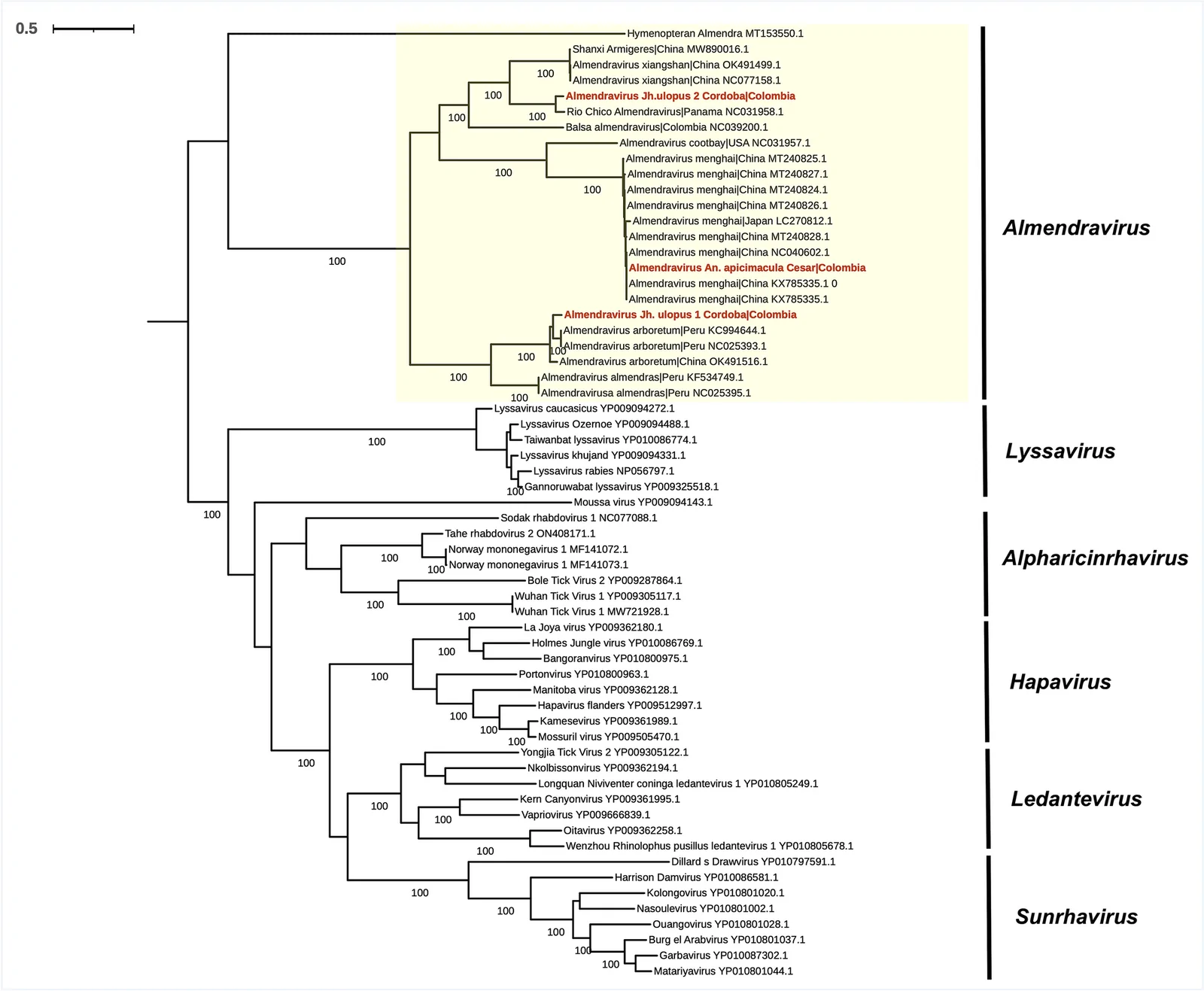
Phylogenetic tree of the L segment of Almendravirus (Rhabdoviridae) from 59 amino acid sequences

**Figure 3:**
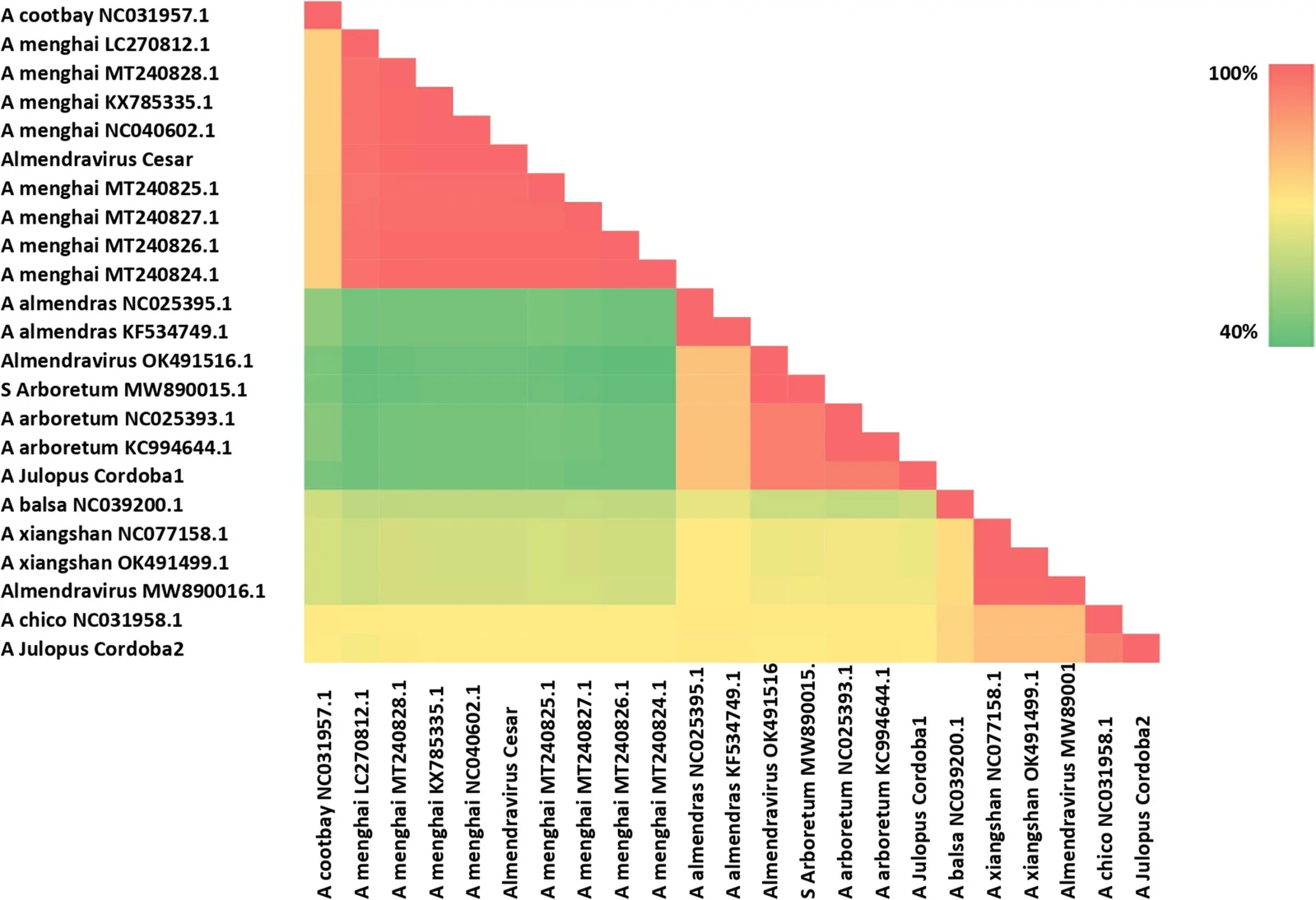
Percent similarity matrix using reference amino acid sequences of the *Almendravirus* genus related to obtained in this study. Created using Clustal Omega of EMBL-EBI (EMBL’s European Bioinformatics Institute) and edited in Excel

In the mosquito pool of *An. apicimacula* from the department of Cesar, a genome named *Almendravirus An. apicimacula* (AAAV) has a genomic sequence of 11,042 bp corresponding to 47,024 reads from 40.878.056 (0.12%), with an average read depth of 638X, and is closely related to the virus MRV China (GenBank: NC_040602.1) (Figs. 2 and 3). Phylogenetic reconstruction of the L protein amino acid sequences was performed using maximum likelihood, and the resulting tree (Fig. 2), inferred from 59 aligned sequences, robustly placed all 3 novel Colombian sequences within the monophyletic genus *Almendravirus* (Rhabdoviridae), which was supported by a bootstrap value of 100% at the node defining the genus (Fig. 2). Specifically, *Almendravirus Jb. ulopus 1* (AJUV 1, Córdoba, Colombia) clustered tightly with *Almendravirus arboretum* (ABTV, Peru; GenBank: KC994644.1 and related sequences) within a highly supported subclade (bootstrap support 100% at the node uniting AJUV 1 with ABTV and closely related Peruvian/Chinese arboretum-like sequences). Similarly, *Almendravirus Jb. ulopus 2* (AJUV 2, Córdoba, Colombia) formed a strongly supported sister group with *Almendravirus chico* (RCHV, Panama; GenBank: NC031958.1) and the Colombian Rio Chico-like sequence (bootstrap 100% at the key node grouping AJUV 2, RCHV, and the additional Colombian entry). These groupings are fully consistent with the high amino acid sequence identities observed in the L protein (91.36% for AJUV 1–ABTV and 91.09% for AJUV 2–RCHV). In contrast, the sequence from *An. apicimacula* (AAAV, Cesar, Colombia) branched basal to the clade containing multiple *Almendravirus menghai* (MRV) isolates from China and Japan (bootstrap support 99% at the node separating the MRV core clade from AAAV and other basal almendraviruses). The node directly supporting the association of AAAV with the MRV-containing lineage received 99% bootstrap support, although AAAV itself occupies a distinct position with clear branch length separation, reflecting its lower sequence identity (~51.98% in L) and suggesting substantial evolutionary divergence. All major internal nodes within Almendravirus exhibited bootstrap values ≥ 99–100%, providing strong statistical support for the inferred relationships and genus monophyly. No topological instability was observed in the key clades relevant to the novel sequences.

The genomic organization of AJUV 1, AJUV 2, and AAAV followed a typical rhabdovirus pattern. Analysis of the intergenic regions (IGR) revealed conserved transcription regulatory sequences (TRS). A putative transcription termination/polyadenylation signal (5′-GAAAAAAA-3′ in the cDNA) was identified at the end of the ORFs (M, G, and L genes), followed by short intergenic spacers and a conserved transcription initiation signal (5′-ACA-3′). These signals were consistent across the three viruses, confirming a compact and functional transcriptional organization characteristic of the *Almendravirus* genus.

The *Almendravirus* N ORF encodes polypeptides of 435 aa (49.7 kDa), 439 aa (48.4 kDa), and 428 aa (48.1 kDa) in AJUV 1, AJUV 2, and AAAV, respectively. BLASTp analysis showed that the AJUV 1 sequence was 94.7% similar to the N protein of ABTV. The AJUV 2 sequence showed 95.9% similarity to the N protein of RCHV. The AAAV N protein shared 35.85% similarity with MRV. The P ORF-encoded proteins were 280 aa (32.5 kDa) and 214 aa (24.7 kDa) in AJUV 1 and AJUV 2, respectively. BLASTp analysis revealed that the amino acid sequence of AJUV 1 showed 72.04% similarity with the P protein of ABTV, and the amino acid sequence of AJUV 2 showed 76.06% similarity with the P protein of RCHV.

The M ORFs for AJUV 1 and AJUV 2 encode polypeptides of 160 aa (AJUV 1, 19.34 kDa; AJUV 2, 19.26). The amino acid sequence of AJUV 1 showed 94.97% similarity with that of the M protein of ABTV, and that of AJUV 2 was identical to that of RCHV. The P and M ORFs of AAAV encode polypeptides of 245 aa (28.75 kDa) and 172 aa (20.3 kDa), respectively. BLASTp analysis revealed that these proteins shared 56.56% and 67.84% identity, respectively, with the P and M proteins of *Almendravirus* sp. 'chester' (GenBank: OZ335331.1), a strain recently identified in the United Kingdom. Furthermore, the M proteins were analyzed for the presence of late-budding domains (L-domains). While the canonical PPPY and PSAP motifs common in vertebrate rhabdoviruses were not identified, the preservation of the M protein’s structural characteristics suggests the use of alternative motifs or mechanisms for viral budding, as previously reported for other insect-specific rhabdoviruses (ISVs).

G ORF-encoded polypeptides of 477 aa (55.8 kDa), 448 aa (51.6 kDa), and 445 aa (52.17 kDa) were identified in AJUV 1, AJUV 2, and AAAV, respectively. pBLAST analysis showed that the AJUV 1 sequence was 84.66% similar to the G protein of ABTV. The AJUV 2 sequence was 80.76% similar to the G protein of RCHV, and AAAV was 33.82% similar to the G protein of MRV. Despite the high sequence divergence observed in the AAAV G protein, comparative structural analysis and multiple sequence alignment revealed 12 highly conserved cysteine residues (CI to CXII) distributed throughout the ectodomain. The conservation of these residues, which are essential for forming disulfide bonds and maintaining glycoprotein folding, reinforces the classification of these sequences within the genus *Almendravirus*.

The U1 ORF was identified in all three Colombian sequences. In AJUV 1, the U1 ORF encodes a polypeptide of 73 amino acids (8.92 kDa) with 56.16% identity to the ABTV U1 protein. For AJUV 2, the U1 ORF consists of 51 amino acids (6.08 kDa) and shares 72.55% identity with the RCHV U1 protein. Although the AAAV U1 protein (51 aa, 5.93 kDa) showed no primary sequence homology to known rhabdoviruses, secondary structure analysis predicted a conserved membrane-bound topology. Specifically, AAAV-U1 features a short extracellular N-terminus (aa 1–5), a transmembrane-spanning helix (aa 6–27), and a cytoplasmic C-terminal tail (aa 28–51). This structural configuration is consistent with the viroporin-like proteins characteristic of the *Almendravirus* genus.

**Figure 4:**
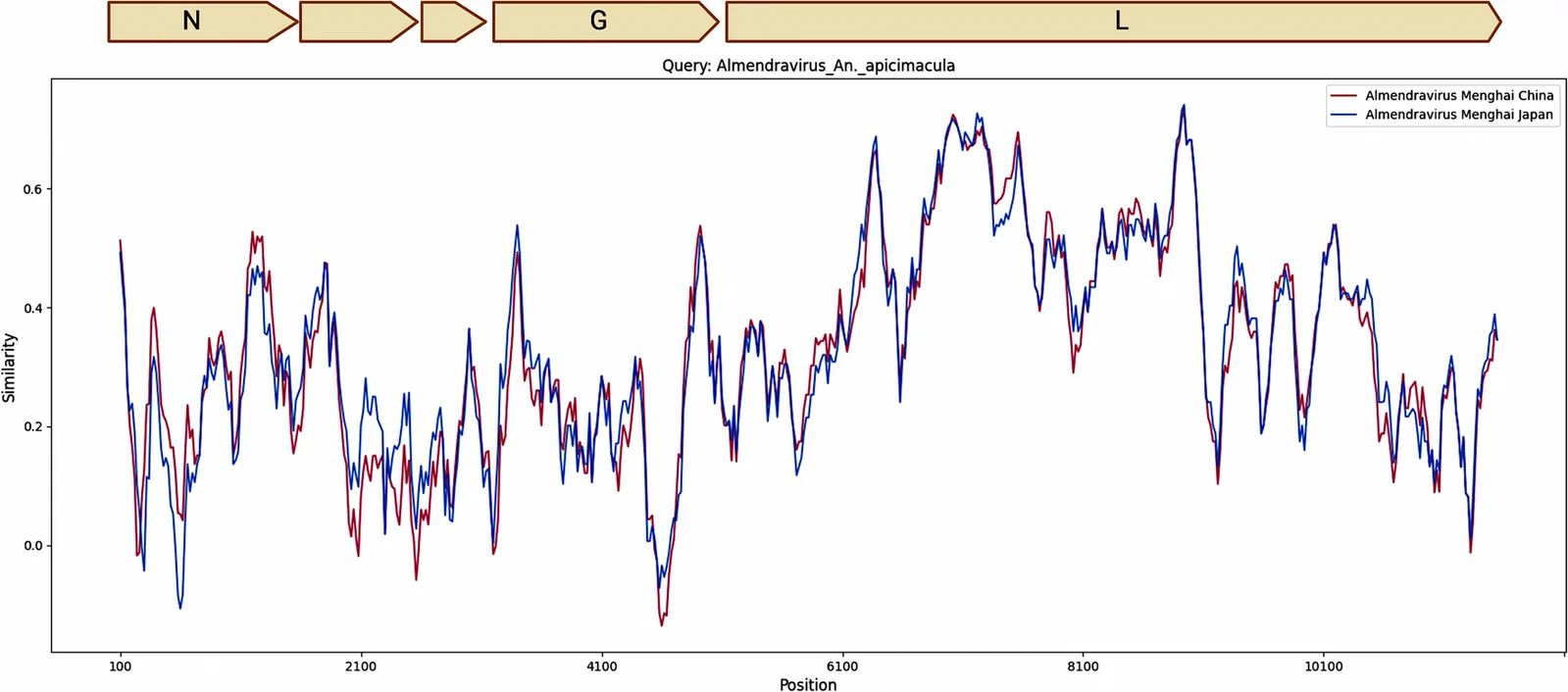
Analysis of genomic divergence between *Almendravirus An. apicimacula* and two phylogenetically close Almendravirus strains using the SimPlo +  + program

**Figure 5:**
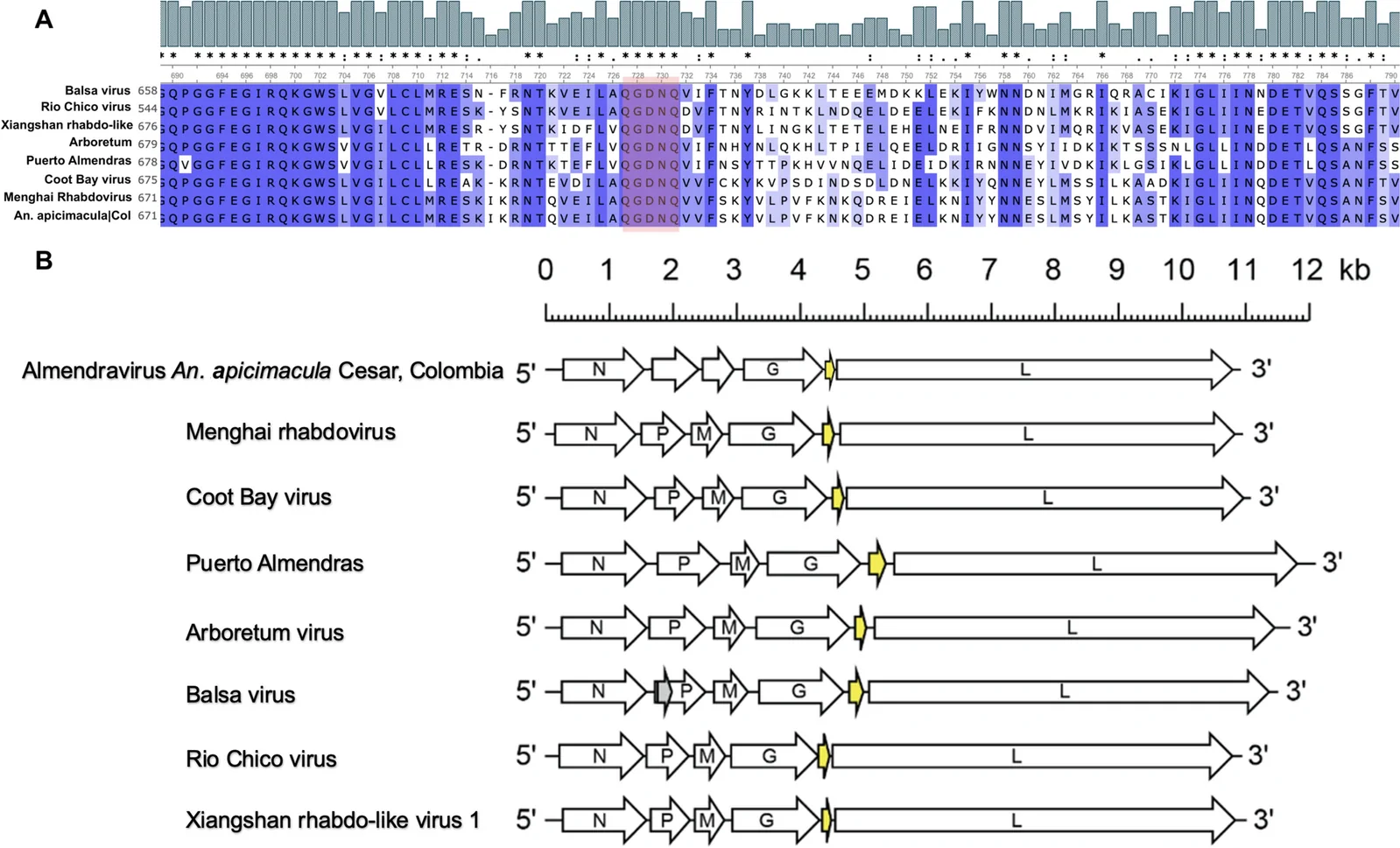
Structural and conserved comparison of Almendravirus. **A** Alignment of amino acids of the L protein (RdRp of negative ssRNA viruses with nonsegmented genomes catalytic domain profile). Highly conserved consensus sequence (GDNQ) is highlighted in red. **B** Schematic of the Almendravirus genomes shown in reverse polarity (positive sense). N, P, M, G, U1 and L represent ORFs encoding structural proteins. U1 ORFs (yellow) encode class 1a viroporin-like proteins. An alternative ORF (Px) in the P gene of the Balsa virus (grey) is also shown (modified from [6])

The L ORF encode polypeptides of 2063 aa (241.18 kDa), 2063 aa (239.54 kDa), and 2055 aa (237.6 kDa) in AJUV 1, AJUV 2, and AAAV, respectively. The AJUV 1 sequence showed 91.36% similarity with the L protein of ABTV. The AJUV 2 sequence showed 91.09% similarity with the RNA-dependent RNA polymerase of RCHV, and AAAV showed 51.98% similarity with the L protein of MRV (Table 2). The L proteins of all three viruses contained highly conserved functional domains characteristic of the Mononegavirales RNA-directed RNA polymerase. Specifically, we identified the RNA-directed RNA polymerase catalytic domain (aa 50–1100), the Mononegavirales mRNA-capping domain V (aa 1000–1400), and the virus-capping methyltransferase connector domain (aa 1300–1550). Additionally, the C-terminal region harbored the RNA-directed RNA polymerase L methyltransferase domain and the Mononegavirus L protein 2-O-ribose methyltransferase (aa 1600–1900). Genomic divergence analysis revealed that the AAAV sequence was highly divergent compared with the MRV sequences from China and Japan (Fig. 4). However, the AAAV genome was found to share ORFs and size, and these conserved domain motifs closely with all members of the *Rhabdoviridae* family (Fig. 5).

**Table 2:** Comparison of Almendravirus L segment proteins based on amino acid sequences

Sequence name	Genome length [Kb]	Protein length [aa]						Genbank phylogenetic similarity of segment L
		Weight [kDa]						
		Position in the genome						%
		N	P	M	G	U1	L	Country
*Almendravirus Jb. ulopus 1* [AJUV 1]	11,631	435	280	160	477	73	2063	YP_009094383.1 91.36% Peru
		49.7	32.5	19.34	55.8	8.82	241.18	
		282–1586	1626–2465	2617–3096	3243–4673	4740–4958	5034–11222	
*Almendravirus Jb. ulopus 2* [AJUV 2]	11,128	439	214	160	448	51	2063	YP_010229226.1 91.09% Panama
		48.4	24.7	19.26	51.6	6.08	239.54	
		215–1531	1564–2205	2279–2758	2824–4167	4204–4356	4400–10588	
*Almendravirus An. apicimacula [AAAV]*	11,042	428	245	172	445	51	2055	QSL97693.1 51.98% China
		48.1	28.7	20.3	52.17	5.93	237.6	
		240–1523	1657–2391	2446–2961	3044–4378	4428–4580	4577–10741

## Discussion

This is the first study to report the presence of *Almendravirus* in *Jb. ulopus* and *An. apicimacula*. In *Jb. ulopus*, new genomic sequences related to ABTV from Peru and RCHV from Panama were detected. A genomic sequence related to the MRV, reported in China, was also detected in *An. apicimacula*.

The identification of three new *Almendravirus* sequences in mosquito species from the Colombian Caribbean highlights the importance of entomovirological surveillance in regions with high biodiversity and complex vector ecosystems. AJUV-1 and AJUV-2 were detected in a single pool of *Jb. ulopus* and AAAV in *An. apicimacula* provides new insights into the virome of these mosquito species and their potential roles in arbovirus ecology.

Phylogenetic analysis of the L protein further supports these relationships and the evolutionary divergence observed. The robust clustering of AJUV 1 with arboretum virus (ABTV) [14] and AJUV 2 with Rio Chico virus (RCHV) [13], both supported by 100% bootstrap values at their respective nodes, confirms their close relationship with previously described South American almendraviruses. In contrast, AAAV occupies a distinct basal position relative to the *Almendravirus menghai* (MRV) clade, with 99% bootstrap support at the node separating it from the core MRV lineage [15]. This strong statistical support, combined with the low amino acid identity in the L protein (~51.98%), aligns with the International Committee on Taxonomy of Viruses (ICTV) criteria for delineating a putative new rhabdovirus species [41]. The high bootstrap values (≥ 99–100%) across major nodes within the genus *Almendravirus* reinforce the monophyly of the group and validate the phylogenetic placement of the novel Colombian sequences.

*Jb. ulopus* is a mosquito species with limited documentation of its vector competence. However, its presence in diverse ecological niches suggests potential interactions with other pathogens. The detection of AJUV 1 and AJUV 2 in the same mosquito pool represents a possible natural co-infection event in *Jb. ulopus*. This finding suggests that *Jb. ulopus* can simultaneously support the replication of multiple *Almendravirus* lineages, potentially acting as an evolutionary hotspot for these viruses in the Colombian Caribbean.

*An. apicimacula* is distributed across several countries in Central and South America, including Colombia. Although its role in malaria transmission has not been well established, its presence in endemic regions suggests its potential epidemiological importance [42, 43]. The identification of AAAV, which exhibits 51.98% similarity with the L protein of MRV, in *An. apicimacula* adds to the growing evidence of diverse viral communities within *Anopheles* mosquitoes [44]. A recent systematic review highlighted the presence of various viruses in *Anopheles* species, emphasizing their potential public health implications [45].

The detection of these *Almendravirus* sequences contributes to a broader understanding of mosquito-associated viruses in Colombia and other regions. Previous studies have highlighted the rich diversity of insect-specific viruses (ISVs) in mosquito populations and their potential to modulate arbovirus transmission. For instance, research has shown that ISVs can interfere with the replication of pathogenic arboviruses within mosquito hosts, thereby affecting their transmission potential [46]. Therefore, characterizing the virome of mosquito species, such as *Jb. ulopus* and *An. apicimacula,* is crucial for developing comprehensive vector control strategies and anticipating the emergence of novel arboviruses.

In 2014, two new viruses were isolated in cell culture from pools of female mosquitoes *Psorophora albigenu* and *Ochlerotatus fulvus* from Peru: Arboretum virus (ABTV) and Puerto Almendras virus (PTAMV), both classified as viruses belonging to the *Rhabdoviridae* family [14]. In 2017, *Almendravirus* was proposed as a new genus belonging to the *Rhabdoviridae* family [13]. In 2020, ABTV was detected in *Ae. albopictus* in Switzerland [47] and was detected in the RNA extracts of *Culiseta longiareolata* in Marcella, France, in 2021 [48]. In 2023, *Almendravirus almendras* was detected in *Ae. albopictus* in Northern Brazil [49]. More recently, sustained circulation of novel almendraviruses derived from *Aedes albopictus* was documented in urban parks over two consecutive years (2023–2024), further highlighting the persistence and urban adaptation of these viruses in this widespread vector [50]. New *Almendravirus* sequences have been reported in three countries across three continents [51]. In addition to the present study, two new sequences were obtained from *Jb. ulopus* mosquitoes, phylogenetically related to ABTV and RCHV, and one sequence with < 90% similarity to the L protein of the MRV obtained from *An. apicimacula* mosquitoes, were reported (Table 3).

**Table 3:** Reports of Almendravirus sequences found in mosquitoes (modified from [38])

Viruses name	Country/year	Host	Total length/contig
AJUV 1	Colombia*/2024	*Jb. ulopus*	11,631
AJUV 2	Colombia*/2024	*Jb. ulopus*	11,128
AAAV	Colombia*/2024	*An. apicimacula*	11,042
*Almendravirus almendras*	Brazil/2023	*Aedes albopictus*	11,884
*Almendravirus arboretum*	Francia/2021	*Culiseta longiareolata*	431–4126
SXABTV	China/2019	*Armigeres subalbatus*	11,48
SXARV	China/2019	*Armigeres subalbatus*	9,59
*Almendravirus menghai* (GD18010)	China/2018	Midges	10,744
*Almendravirus menghai* (GD18008)	China/2018	Midges	10,744
*Almendravirus menghai* (GD18005)	China/2018	Midges	10,744
*Almendravirus menghai* (GD18004)	China/2018	Midges	10,743
*Almendravirus menghai* (GD18003)	China/2018	Midges	10,744
*Almendravirus menghai* (kunoichi)	Japan/2018	*Ae. albopictus* cultured cell line C6/36	10,777
*Almendravirus chico*	Panama/2013	*An. trianulatus*	10,735
*Almendravirus Coot Bay*	USA/2013	*An. quadrimaculatus*	10,869
*Almendravirus balsa* (CoB 84)	Colombia/2013	*Ps. albigenu*	11,286
*Almendravirus balsa* (CoB 76)	Colombia/2013	*Ps. albigenu*	11,287
*Almendravirus menghai* (Menghai)	China/2010	*Ae. albopictus*	10,744
*Almendravirus almendras*	Peru/2009	*Ochlerotatus fulvus*	11,876
*Almendravirus arboretum*	Peru/2009	*Ps. albigenu*	11,482

The sequence detected in the present study for *An. apicimacula* shares 51.98% similarity with the L protein of reported MRV sequences in China. MRV was first isolated in 2010 from *Ae. albopictus* in Menghai, Yunnan, China [15]. In addition, in 2010, MRV was detected in *Culex tritaeniorhynchus* and *Ae. albopictus* from the Lancang and Un rivers in China [52]. In contrast, another study showed that when MRV and Zika virus (ZIKV) were grown simultaneously in C6/36 cells of the ECACC collection, the growth kinetics of ZIKV decreased [52]. This suggests the suppression of the growth of some arboviruses that infect humans.

In contrast, several studies have identified viruses in insects without specifying the role of these viruses in the vector. Viruses assigned to different species within the genus *Almendravirus* have several characteristics: a minimum amino acid sequence divergence of 5% in the N proteins, a minimum amino acid sequence divergence of 10% in the L protein, a minimum amino acid sequence divergence of 15% in the G proteins, and significant differences in the organization of the genome, as evidenced by the number and location of the ORFs, which can be distinguished in viral neutralization tests. They occupy different ecological niches, as evidenced by differences in their hosts and arthropod vectors [8]. Our study identified viral sequences with low similarity to *Almendravirus menghai* sequences. However, we propose that it is a new rhabdovirus species, considering that the L segment has a low percentage of similarity with other rhabdoviruses.

The viruses detected in this study presented ORFs that coded for the N, P, M, G, U1, and L proteins of the *Almendravirus* genus. The L segment has a conserved amino acid sequence in the coding region among all proteins [15]. The coding region of the L gene is located in the RdRp, which determines the replication of the viral genome [14]. Sequences with a similarity of less than 90% in the coding region of the L segment can be considered as new species of rhabdovirus [53]. The L segments of AJUV 1 and AJUV 2 contained all motifs of the conserved domains of the RdRp members of *Almendravirus* [54]. AJUV 1 and AJUV 2 were also found in the methyltransferase domain of the Mononegavirales RNA polymerase enzymes [55].

Despite the low sequence similarity, functional domain analysis confirmed the presence of a nucleocapsid-associated domain in the N protein, consistent with its role in binding genomic RNA within the ribonucleoprotein complex (RNP), as described previously [8]. Conserved domains were detected in the G protein. A detailed mapping of the AAAV G protein revealed the presence of 12 highly conserved cysteine residues (CI–CXII). The preservation of these residues, despite the low overall amino acid similarity, indicates that the fundamental structural scaffold and disulfide bridge formation required for glycoprotein stability are maintained [51, 56], further supporting its inclusion in the *Almendravirus* genus.

The absence of canonical L domains (PPPY and PSAP motifs) in the M protein of AJUV 1, AJUV 2, and AAAV is a notable finding. In mammalian-infecting rhabdoviruses, these motifs are essential for viral budding through interaction with the host ESCRT pathway machinery, such as TSG101 [57]. However, this absence has been previously documented in other insect-specific rhabdoviruses (ISVs) and members of the *Almendravirus* genus. This suggests that these viruses may employ alternative budding strategies or motifs that have yet to be characterized, further supporting the hypothesis that Almendraviruses are highly specialized for replication in arthropod hosts and lack the molecular tools required for interaction with vertebrate cellular export systems.

Characterization of the AAAV genome revealed substantial divergence from known members of the *Almendravirus* genus, particularly in structural proteins. The nucleoprotein (N), glycoprotein (G), and RNA-dependent RNA polymerase (L) exhibited low amino acid similarity with their closest counterparts, ranging from 33.82% to 51.98%. Notably, the phosphoprotein (P) and matrix protein (M) did not show significant homology to any other known rhabdovirus, highlighting a specific evolutionary link between AAAV and this European lineage within the *Almendravirus* genus [13, 14, 58, 59]. In addition, the U1 protein was present, notably in the highly divergent AAAV. The genomic position and predicted molecular weight of the U1 gene were consistent with the viroporins described for other members of the genus [8, 13]. Furthermore, the conservation of the structural architecture over primary sequence identity highlights the evolutionary pressure to maintain the role of the U1 protein in the *Almendravirus* life cycle.

The genetic divergence observed across the entire AAAV genome, coupled with its 100% sequence coverage, indicates that its unique profile is not a result of technical artifacts or incomplete data. Instead, this divergence likely reflects a long-standing evolutionary process influenced by geographic isolation. Given that its closest relatives are found in Asia and Europe, the genetic distance observed in Colombia suggests significant evolutionary pressure over time and a potential lack of genomic data for intermediate lineages in the Americas. Consequently, the results support the hypothesis that AAAV represents a novel species within the *Rhabdoviridae* family, according to the International Committee on Taxonomy of Viruses [41].

## Conclusions

The findings of this study demonstrate remarkable viral diversity in Neotropical mosquitoes. The detected viruses showed high genetic variability. The newly identified viral strains can be further analyzed to assess their pathogenic potential and interactions with other viruses within the vectors. Taken together, these findings strongly suggest that AAAV constitutes a previously unrecognized rhabdovirus, expanding the known diversity of this group and highlighting the utility of metagenomic approaches for virus discovery in underexplored species.

## Data Availability

Sequences are available in the Sequence Read Archive (SRA) with accession number PRJNA1251058.
